# Some Experiments with Calomel as a Diuretic

**Published:** 1889-10

**Authors:** J. G. Edgren, L. G. Sellstedt


### Translations.

### SOME EXPERIMENTS WITH CALOMEL AS A DIURETIC.

By Prof. J. G. EDGREN.

From the original Swedish, in *Hygiea*, by Mr. L. G. Sellstedt.

It is known that calomel has of late been highly recommended as an excellent agent in increasing diuresis in cardiac ascites and anasarca. Persons with in sufficient heart-energy, and consequent exudations into the serous cavities of the body and the subcutaneous areolar tissues, have, in a short time, had these fluids lessened or entirely removed by calomel treatment. In by far the greatest part, this has been the result of a considerable increase in the flow of urine, which took place the second or third day after the commencement of the treatment. The usual dose has commonly been twenty centigrams three times a day, and but a few days' treatment has sufficed, in a marked degree, to increase diuresis, and thus give the patient great relief, even if this, in the nature of the case, could only be temporary. Opportunity to prove these statements has richly been found in the Seraphim Hospital of late, and it may possibly be of interest to describe a few cases thus treated with calomel. To this end, I will begin with a case when the working of calomel was particularly striking, while that of digitalis showed itself impotent in any considerable degree to augment the diuresis. This case is even of interest in that the calomel treatment was continued longer (eleven successive days) than in general had been the case, and without exhibiting the smallest symptom of poisoning:

Anna W., 66 years old, widow, was received at the Seraphim Hospital the 7th September, 1888.

The father of the patient died, at the age of 84, of old age, the mother, at 57, of consumption; even her maternal grandmother seems to have died of consumption. In her home, she labored hard, but in other respects was comfortably situated. She had not used alcoholic stimulants, but had drank a great deal of coffee. She had the measles when a child, was well afterwards until she was fifty years of age, when she lay sick for seven weeks with what, according to her statement, must have been typhoid fever. She has never had fever and-ague, rheumatism, or lues. Her menses commenced at 17, and have always been regular. She had the first sign of her present illness three years since. After taking cold, she had pains in her left side, in the region of the heart, palpitation of the heart; the pains went away, but the palpitation continued. About midsummer 1877, she discovered, for the first time, that her left foot was swollen. The swelling spread up the leg, and appeared even in the other foot and leg, so that in about eight days it had reached her waist. The physicians consulted informed her that she had heart-failing. Medicine was prescribed, and the swelling disappeared in a few days, reappeared after a fortnight, but vanished anew on applying the same remedies. Afterwards, the patient was, with the exception of the palpitation, tolerably smart till Christmas eve, when the legs began to swell again, and soon invaded even the abdomen. In the beginning, the swelling disappeared at night, but soon became permanent. During the Spring, the swelling increased more and more, and at last she applied for admission to the Seraphim Hospital. Upon entering the hospital, her condition was mainly the following:

The patient was much reduced in flesh, the abdomen enormously distended, containing free liquid, and measured around the navel 110 centimeters. Edema of the feet, above the ankles, reaching upward on the legs, the waist, and the walls of the abdomen, and the front of the thorax, above the nipples. The precordium somewhat bent forward, pulsations in the third, fourth, fifth, and sixth interstice. Plain, positive, systolic vein-pulse, not only in the veins of the neck, but in the network of veins in front of the thorax, as far as its termination. The beat of the point of the heart was felt in the sixth rib-interstice, in the forward part of the line of the axilla. The upper boundary of the heart-pressure was the third rib, and downwards, at the sixth; to the left, as far as the forward line of the axilla; to the right, it extended to a depression[1] which occupied the greater part of the half of the thorax. Over the point of the heart was felt a weak systolic tremor (*fremissement*). In auscultation, both heart-tones were heard; after the first, a prolonged blowing sound, plainest at the point and to the left, at the lower end of the sternum, but was perceptible even at the base. The diastolic sound was fairly clear, though some slight obscurity and faint scraping sound was sometimes perceived. The action of the heart was irregular, the arteriæ radialis somewhat stiff, but not pearl-stringlike. Over the fore-part of the right lung, a depression from the upper edge of the third rib; on the back part, a depression from angulus scapulæ, on both sides, downwards. The respiratory sound, weak where the depression commences, and almost inaudible farther down; sparse rhonchi and moist ralls. Pectoral fremitus extremely weak over the lower parts of the lungs. Urine, acid, sp. gr. 1020, has trace of albumen; amount, 400 cubic-centimeters per day (twenty-four hours).

The result of examination indicated that the patient suffered from organic heart-failure, insufficiency of the mitral valves, possibly complicated with stenos in ostium venos sinistrum, together with insufficiency in valvula tricuspidalis, insufficiency of the muscles of the heart, and consequent edema, ascites, and hydro-thorax.

The treatment and its results I have arranged in the following table:

| Date. | | Treatment. | Quantity of Urine. Cubic-centimeters. | Abdominal Size. Centimeters. |
|---|---|---|---|---|
| September | 8 | } Digitalis (8–10) . . . . . . . . . . . . | 400 | 110 |
| " | 9 | Digitalis . . . . . . . . | 500 | . . |
| " | 10 | . . . . . . . . . . . . . | 300 | . . |
| " | 11 | . . . . . . . . . . . . . | 600 | . . |
| " | 12 | . . . . . . . . . . . . . | 900 | . . |
| " | 13 | . . . . . . . . . . . . . | 900 | . . |
| " | 14 | . . . . . . . . . . . . . | 900 | 110 |
| " | 15 | } Digitalis (15–16) . . . . . . . . . | 700 | . . |
| " | 16 | . . . . . . . . . . . . . | 1,200 | . . |

1. The word which is here translated depress means to suppress or smother.—Tr.

| Date. | | Treatment. | Quantity of Urine. Cubic-centimeters. | Abdominal Size. Centimeters. |
|---|---|---|---|---|
| September | 17 | . . . . . . . . . . . . . | 1,000 | . . |
| " | 18 | . . . . . . . . . . . . . | 800 | . . |
| " | 19 | . . . . . . . . . . . . . | 500 | . . |
| " | 20 | Calomel, 0.20, 3 times daily, (19–21) | 600 | . . |
| " | 21 | . . . . . . . . . . . . . | 2,700 | 105 |
| " | 22 | . . . . . . . . . . . . . | 2,600 | . . |
| " | 23 | . . . . . . . . . . . . . | 900 | . . |
| " | 24 | . . . . . . . . . . . . . | 800 | . . |
| " | 25 | Digitalis (24–26) . . . . . . . . | 700 | . . |
| " | 26 | . . . . . . . . . . . . . | 800 | . . |
| " | 27 | . . . . . . . . . . . . . | 700 | 106 |
| " | 28 | . . . . . . . . . . . . . | 1,600 | 104 |
| " | 29 | . . . . . . . . . . . . . | 3,000 | 101 |
| " | 30 | . . . . . . . . . . . . . | 1,800 [1] | . . |
| October | 1 | . . . . . . . . . . . . . | 2,900 [1] | 99 |
| " | 2 | Calomel, 0.20, 3 times daily, (Sept. 27–Oct. 7) | 3,100 | 96 |
| " | 3 | . . . . . . . . . . . . . | 3,300 | . . |
| " | 4 | . . . . . . . . . . . . . | 2,600 | 90 |
| " | 5 | . . . . . . . . . . . . . | 2,500 | . . |
| " | 6 | . . . . . . . . . . . . . | 2,400 | 83 |
| " | 7 | . . . . . . . . . . . . . | 2,400 | . . |
| " | 8 | . . . . . . . . . . . . . | 1,800 | 78 |
| " | 9 | . . . . . . . . . . . . . | 1,500 | . . |
| " | 10 | . . . . . . . . . . . . . | 1,500 | 76 |
| " | 11 | . . . . . . . . . . . . . | 1,100 | . . |
| " | 12 | . . . . . . . . . . . . . | 1,400 | . . |
| " | 13 | . . . . . . . . . . . . . | . . | 75 |

When the patient was discharged, the 13th of October, the edema, ascites, and hydro-thorax were completely absent, the action of the heart regular, the point of the heart somewhat in advance of the forward-line of the axilla; weak systolic second sound at the point; accentuated second tone over the pulmonalis. Vein pulsation still visible, but much weaker; abdominal walls shrunk and sharply wrinkled, the liver easily felt, no marked changes in the same.

In this case, the use of digitalis did not succeed in bringing about a sufficient diuresis. It is true that the action of the heart became more regular, and an increase of urine was exhibited, but too slight to be of consequence. On the other hand, the quantity of urine increased very considerably with the first use of calomel, 60 centigrams daily, during three days. A new attempt with digitalis gave no result. When now calomel, for the second time, was employed, it was continued eleven days with the same daily dose, with the result that the diuresis was enormously increased, and, in consequence thereof, the

1. During these days, the patient had diarrhea, and the urine could not be perfectly measured transudations everywhere disappeared. During the whole of this time, the patient exhibited no trace of toxical effect, and only during two days a slight diarrhea appeared.

Several other cases may be cited where fluids have collected, as well in the great serous cavities as in the areolar tissue, by reason of the insufficient working of the heart. Thus there is, at present, the case of a boy, nine years of age, indicating insufficiency and stenos in the mitral valves, ascites, and considerable edema of the lower extremities. At his admission, the measure round the abdomen was eighty centimeters, and the quantity of urine during the first days of his stay ranged from 150 to 400 cubic-centimeters; nay, sank as low as fifty cubic-meters a day. After three days' exhibition of calomel, in doses of ten centigrams three times daily, the quantity of urine rose to 2,110 and 2,900 centimeters a day. Now the medicine was discontinued for two days, and again calomel was resorted to, with the same doses as before. Under this, the quantity of urine was between 1,500 and 2,000 cubic centimeters per day for six days. The increased diuresis resulted in an important abatement of the dyspnea and the edema of the lower parts of the body; and the abdomen, in a fortnight, diminished from eighty to sixty-eight centimeters. Even in cases where no clinical signs of valve failures were found, but insufficiency in the power of the heart may have been due to degenerative processes in the muscular structure, independent of valve failure, calomel has been proved a most powerful diuretic. A watchful care of the patient must, however, be exercised. Signs of salivation may show themselves in some patients after taking a few twenty-centigram doses. Thus, a girl, 26 years of age, who was cared for at the Seraphim Hospital from the 12th of September till 18th of October, with plainly exhibited symptoms of insufficiency and stenos in the mitral valves, already, after four days' treatment, (0.20 x 3 daily) exhibited toxical symptoms (salivation and tenderness in the gums), when, of course, the remedy was discontinued. The quantity of urine rose from 400 cubic-centimeters a day, before the treatment, to 1,000, 1,800, 2,400 cubic-meters, and the abdominal measure lessened from eighty-six to seventy-six centimeters. The diuretical action of calomel is, therefore, very plain, and, so far as I can see, constant, where the abnormal collection of water depends exclusively on insufficiency in the muscular structure of the heart. If, on the contrary, obstructions in the portal system, with or without heart-failing, be the cause of the ascites and edema, it seems the diuresis cannot be increased, at least in the same degree, by calomel.

Several other cases, in support of this opinion, are minutely described by the learned author, which, for want of space, must be omitted in this number.

It is worthy of note, however, that in the experimental treatment with calomel, a successful increase of diuresis has not failed to follow its use, when the ascites or other dropsical symptoms were due to heart-failure or valvular insufficiencies, and that, in all these cases, its superiority over digitalis was established. When, however, the edema, ascites, or other kindred symptoms, were the result of disease of the liver or the portal circulation, neither calomel nor digitalis were found effective. In support of this, an interesting account is given, where a middle-aged woman was under treatment for dropsical affection. Though, in this case, there were symptoms indicating valvular insufficiency, both calomel and digitalis were found impotent in producing increase of diuresis. Hydro-thorax supervened, and the patient died. The autopsy disclosed cancer of the liver. This organ had not been examined, on account of the distended state of the abdomen, and tapping had been deferred, owing to the low state of the patient. The heart was found in normal condition, and the valves in good working order.

In conclusion, the author remarks: "During the use of calomel, poisoning has very seldom supervened. Of all the cases which I have thus treated, the one alluded to above is the only one which has exhibited a slight swelling of the gums and any salivation. A slight diarrhea, consisting of some loose movements during a couple of days, has, however, a few times, been observed. Any deleterious effect on the action of the heart I never have noticed; digitalis has always been given two or three days—a longer or shorter time—before resorting to the calomel. Still, it may be necessary to watch over the patient during the whole time of the calomel treatment, so that the remedy may be discontinued at the first sign of salivation.

The results my experiments with calomel, as a diuretic, have given, seem to me to be comprised in the following statement:

1. Calomel is a very sure and quickly-acting diuretic in cardial hydrops.

2. If calomel does not increase the diuresis in a person suffering from ascites and anasarca, whose condition is not so low that, taken all in all, every medicinal treatment must be regarded futile, the probability is that the cause of the watery exudations must be looked for elsewhere than in the heart."

